# Prolonged grief symptoms and lingering attachment predict approach behavior toward the deceased

**DOI:** 10.1002/jts.23124

**Published:** 2025-01-06

**Authors:** Maarten C. Eisma, Thomas A. de Lang, Katerina Christodoulou, Lara O. Schmitt, Paul A. Boelen, Peter J. de Jong

**Affiliations:** ^1^ Department of Clinical Psychology and Experimental Psychopathology University of Groningen Groningen the Netherlands; ^2^ Department of Experimental Psychology University of Groningen Groningen the Netherlands; ^3^ Department of Clinical Psychology Utrecht University Utrecht the Netherlands; ^4^ ARQ National Psychotrauma Centre Diemen the Netherlands

## Abstract

Following the death of a loved one, both approach behaviors related to the deceased (i.e., engagement with feelings, memories, and/or reminders of the deceased) and the avoidance of reminders of the death are theorized to precipitate severe and persistent grief reactions, termed prolonged grief. The “approach‐avoidance processing hypothesis” holds that these behavioral tendencies occur simultaneously in prolonged grief disorder (PGD). We tested this hypothesis using a novel free‐viewing attention task. Bereaved adults (*N* = 72, 81.9% female) completed a survey assessing prolonged grief symptoms, depressive symptoms, and lingering attachment and a free‐viewing task assessing voluntary attention toward pictures of the deceased and combinations of the deceased with loss‐related words (i.e., loss‐reality reminders). A main finding was that participants with higher prolonged grief symptom levels, ρ(70) = .32, *p* = .006, and more lingering attachment, ρ(70) = .26, *p* = .030, showed stronger attentional focus toward pictures of the deceased. No significant association emerged between either prolonged grief symptom levels or lingering attachment and attention toward loss‐reality reminders. The findings suggest that higher prolonged grief symptom levels may be characterized by persisting approach tendencies toward the deceased. Countering excessive proximity‐seeking to the deceased in therapy could be beneficial for bereaved adults who show severe and persistent grief reactions.

Bereavement is the most stressful life event that most people will experience during their lifetime. A minority of bereaved adults experience severe, persistent, and disabling grief, termed *prolonged grief*. Two diagnoses characterized by such grief reactions have been included in the *International Statistical Classification of Diseases and Related Health Problems* (11th rev.; *ICD‐11*; World Health Organization [WHO], 2019) and the *Diagnostic and Statistical Manual of Mental Disorders* (5th ed., text rev.; *DSM‐5*‐*TR*; American Psychiatric Association [APA], 2022) as prolonged grief disorder (PGD). Although these diagnoses share key features (i.e., yearning for the deceased and cognitive preoccupation are core symptoms of both), they are different in several respects (i.e., the timing criteria, number and type of accessory symptoms, and diagnostic algorithms; Eisma, 2023). Experiencing the sudden and/or unnatural death of a loved one is associated with a heightened risk of developing PGD (Djelantik et al., 2020; Doering et al., 2022).

Many grief theorists hold that avoiding painful aspects of a loss, such as memories, objects, and situations, may interfere with the cognitive processing of the loss, thereby leading to prolonged grief reactions (e.g., Boelen, van den Hout, et al., 2006; O'Connor & Seeley, 2022; Shear et al., 2007). In line with such notions, loss‐related avoidance, cognitive avoidance strategies (e.g., thought suppression), and experiential avoidance are positively associated with prolonged grief symptom severity (for a review, see Eisma & Stroebe, 2021). Moreover, interventions that aim to reduce such avoidance tendencies, such as exposure therapy, have been shown to reduce prolonged grief symptom severity both when administered as a standalone treatment and when included as an added treatment element (e.g., Boelen et al., 2007; Bryant et al., 2014; Eisma, Boelen et al., 2015). Reductions in loss‐related avoidance are also a mediator of the effects of cognitive behavior therapy (CBT) on prolonged grief symptoms (e.g., Glickman et al., 2017; Lechner‐Meichsner et al., 2022).

Despite the well‐established role of avoidance in severe grief reactions, some researchers have proposed a role for approach behaviors in the emergence of prolonged grief (e.g., Boddez, 2018; Kakarala et al., 2020; LeRoy et al., 2019). For example, LeRoy et al. (2019) built on Bowlby's (1982) idea that people in one's social environment fulfill attachment‐related needs, such as proximity‐seeking (i.e., seeking closeness and resisting separation), safe haven (i.e., reducing distress and providing support during difficult times) and secure base (i.e., providing a core sense of emotional and psychological security). People in one's social environment are organized in an individual attachment hierarchy wherein one's partner typically fulfills most of the important attachment‐related functions and is on top of the hierarchy, and secondary attachment figures, such as parents, siblings, and friends are lower in the hierarchy (Trinke & Bartholomew, 1997). Following the loss of an attachment figure, the extent to which one succeeds in reorganizing their attachment hierarchy to fulfill attachment‐related needs is proposed to determine their psychological and physiological recovery. However, an individual's inability to direct attachment‐related needs toward new people in their social environment and a strong desire to still use the deceased person for attachment‐related needs can perpetuate rumination about the loss and loss‐related distress (LeRoy et al., 2019).

In line with these ideas, there is evidence that continuing bonds with the deceased, the desire to use a lost person as an attachment figure (often termed “desired attachment” or “lingering attachment”), and approach behavior toward the deceased are related to more severe levels of prolonged grief following interpersonal loss (Boelen, Stroebe, et al., 2006; Eisma et al., 2022; Field et al., 2003). Moreover, interventions to counter persistent and strong continuing bond behaviors, such as reducing the time the bereaved engages in these types of behaviors, are part of some grief‐focused CBTs (Boelen, van den Hout, et al., 2006), although no trials to date have separately examined the effects of this technique on prolonged grief symptoms.

Based on the above, the “approach avoidance processing hypothesis” was formulated. This hypothesis holds that prolonged grief is characterized by the co‐occurrence of approach behaviors toward the deceased and the avoidance of reminders of the separation from the deceased (i.e., *loss reality*; Eisma & Lenferink, 2023). These processes are proposed to be self‐perpetuating, as they increase positive feelings (e.g., loyalty toward the deceased, connectedness with the deceased) and reduce negative feelings that confrontation with the reminders of the loss‐reality may elicit. These co‐occurring behavioral tendencies are proposed to hamper the cognitive processing of the loss (cf. Boelen, van den Hout, et al., 2006; O'Connor & Seeley, 2022), leading to persistent severe grief. In line with this theory, a recent latent class analysis study showed that roughly one third of a sample of bereaved adults demonstrated high odds of behaviors that appear to maintain a focus on the deceased (i.e., proximity‐seeking, rumination, yearning) as well as behaviors indicative of the avoidance of reminders of separation from the deceased and associated emotional experiences (i.e., loss‐related avoidance, experiential avoidance). These participants, in turn, demonstrated higher levels of prolonged grief symptoms and a higher prevalence of probable PGD than those in classes with lower odds of loss‐related approach and avoidance behaviors (Eisma & Lenferink, 2023).

Although Eisma and Lenferink's (2023) study forms an important first step in clarifying the validity of the approach avoidance processing hypothesis, the self‐report methodology used in that study has important limitations. For example, self‐report data is subject to biases (e.g., social desirability), and bereaved people may be unwilling or unable to accurately report on their—at times conflicting—approach and avoidance tendencies. A broadly accepted, face‐valid technique to study approach and avoidance tendencies is the study of overt behavior and attention using laboratory tasks, which is increasingly applied in bereaved samples (e.g., Arizmendi et al., 2023; Eisma et al., 2014; Eisma, Rinck, et al., 2015; Maccallum & Bryant, 2019; Maccallum et al., 2015; Michel et al., 2023; Schneck et al., 2018; Yu et al., 2017). However, the findings from laboratory studies regarding approach and avoidance tendencies in bereaved adults have been mixed, with some providing evidence of loss‐related avoidance tendencies among bereaved people with severe grief reactions (e.g., Eisma et al., 2014; Yu et al., 2015) and others providing preliminary evidence for loss‐related approach tendencies in similar samples (e.g., Maccallum & Bryant, 2019; Maccallum et al., 2015).

These mixed findings appear to be due to methodological variability. Some laboratory techniques solely enable the establishment of attentional interference of stimuli (e.g., Stroop task; Schneck et al., 2018; Michel et al., 2023), whereas other techniques are designed to assess automatic approach and avoidance tendencies (e.g., approach–avoidance task [AAT]; Arizmendi et al., 2023; Maccallum et al., 2015) and still others can assess both automatic and voluntary approach and avoidance tendencies (e.g., dot‐probe task, eye‐tracking tasks; Eisma et al., 2014; Yu et al., 2015). Another point of variability consists of employed stimuli, with studies employing generic loss‐related words, generic loss‐related pictures, names of the deceased, and pictures of deceased persons. When a researcher seeks to use information from these studies to evaluate the approach avoidance processing hypothesis, a notable limitation is that comparatively few attention studies have used specific reminders of the deceased (for notable exceptions, see Maccallum & Bryant, 2019; Mancini & Bonanno, 2012), and still fewer have employed stimuli representing reminders of the separation from the deceased (but see Eisma et al., 2014, Eisma, Rinck, et al., 2015). Moreover, to our knowledge, no studies to date have examined approach behaviors related to the deceased and the avoidance of the loss reality within a single bereaved sample, which makes it difficult to establish whether such tendencies co‐occur in people with elevated levels of prolonged grief symptoms. The present study sought to fill this gap in knowledge.

The primary aim of this study was to test the approach avoidance processing hypothesis. We designed a novel free‐viewing attention task to assess voluntary attention toward the deceased and reminders of loss reality among bereaved adults. We tested two hypotheses and posited that participants with higher levels of prolonged grief symptoms would show (a) relatively stronger attentional focus on the deceased (i.e., approach toward the deceased) and (b) relatively weaker attentional focus on reminders of the loss reality (i.e., avoidance of the loss reality).

A secondary goal was to explore associations between lingering attachment and loss‐related approach and avoidance tendencies, as assessed using the free‐viewing task. Lingering attachment, or the desire to use the lost person for attachment‐related goals, has been proposed to interfere with the grieving process and perpetuate loss‐related distress (LeRoy et al., 2019; Mikulincer & Shaver, 2022). In line with previous laboratory research among individuals who experienced a romantic relationship break‐up (Eisma et al., 2022), we formulated two hypotheses: that stronger lingering attachment would be associated with (a) relatively higher prolonged grief symptom levels and (b) a relatively stronger attentional focus on the deceased.

## METHOD

### Participants

Individuals were eligible to participate if they were 18 years of age or older and had experienced the death of a loved one at least 1 year ago, which is in line with the *DSM‐5‐TR* and *ICD‐11* timing criteria for PGD. Individuals were ineligible to participate if they had a very low level of educational attainment (primary school or less), did not experience the loss of a human (e.g., a pet loss), had current or past psychosis, or experienced strong suicidal ideation or had concrete suicide plans. We recruited 61 participants by contacting respondents from a previous survey who indicated that they were interested in participating in a laboratory study; 13 additional participants were recruited via purposeful sampling from the social networks of two students involved in executing the study. Data from two people were invalid due to software and human error and thus excluded, leaving 72 participants. Table 1 summarizes the sample characteristics.

**TABLE 1:** Sociodemographic and loss‐related characteristics of the sample.

Variable	*M*	*SD*
Sociodemographic characteristics		
Age (years)	49.96	13.94

	*n*	%
Gender identity		
Male	12	16.7
Female	59	81.9
Other	1	1.3
Educational attainment		
College or university	50	69.4
Other than college or university	22	30.6
Loss‐related characteristics		
Time since loss (months)		
12–35	25	34.7
36–60	25	34.7
≥ 60	22	30.6
Gender identity of the deceased		
Male	39	54.2
Female	32	44.4
Other	1	1.3
Deceased person		
Partner, lover, or spouse	25	34.7
Child	12	16.7
Sibling	5	6.9
Parent	20	27.8
Other	10	13.9
Cause of death		
Natural death	60	83.3
Accident	3	4.2
Suicide	9	12.5
Expectation of death		
Expected	28	38.9
Unexpected	35	48.6
Both or neither	9	12.5

*Note*: *N* = 72.

### Procedure

The Medical Ethical Committee of the University Medical Center Groningen approved the current study (NL75661.042.20). All participants provided written informed consent. Individuals who were approached to participate in the study were emailed a letter that included information about the study (e.g., study goals, advantages and disadvantages of participation, information on anonymity and voluntariness) and an informed consent form. People who were interested in participating contacted the researchers by e‐mail, after which a researcher contacted them by phone so the potential participant could ask any questions they had, provide oral informed consent, and complete an eligibility screening; those who were deemed eligible were then scheduled for a laboratory visit at the University of Groningen or Utrecht University.

In the laboratory, participants reread the informational letter, provided written informed consent, and filled out a survey that was programmed in Qualtrics. They then completed three laboratory tasks: an approach avoidance task, an eye‐tracking task, and the free‐viewing attention task that was the focus of the current study; notably, the first two tasks used similar stimuli as the third task. After the tasks, a researcher asked participants about their experiences and explained the research aims, and participants filled in a form to receive €25 (EUR) for their participation as well as reimbursement for travel costs. Participants also received the contact details of an independent psychiatrist, who could refer them to appropriate care in case they felt they needed help. One participant, who did not display clinical levels of prolonged grief symptoms, indicated a need to discuss their emotional response to the procedure but no need for help; she had one meeting with a certified clinical psychologist who was part of the research team. This participant did not indicate the need for a follow‐up meeting. In total, the laboratory visit took approximately 1 hr to complete.

#### Free‐viewing attention task

We designed a novel free‐viewing attention task in OpenSesame, an experiment‐building software based on Python (Mathôt et al., 2012), to assess voluntary attention toward the deceased and loss reality reminders. Before the laboratory visit, participants were asked to send two high‐quality digital pictures of their deceased loved one. To protect participants’ privacy, we asked them to send the files to a secure file‐sharing system (WeTransfer), which gives the recipient access for a limited period, after which the files are deleted. Participants were also asked to remove any details that could reveal the identity of the deceased person (e.g., a person's name in the file name). Moreover, they were informed that the pictures would be deleted immediately after participation.

**FIGURE 1:**
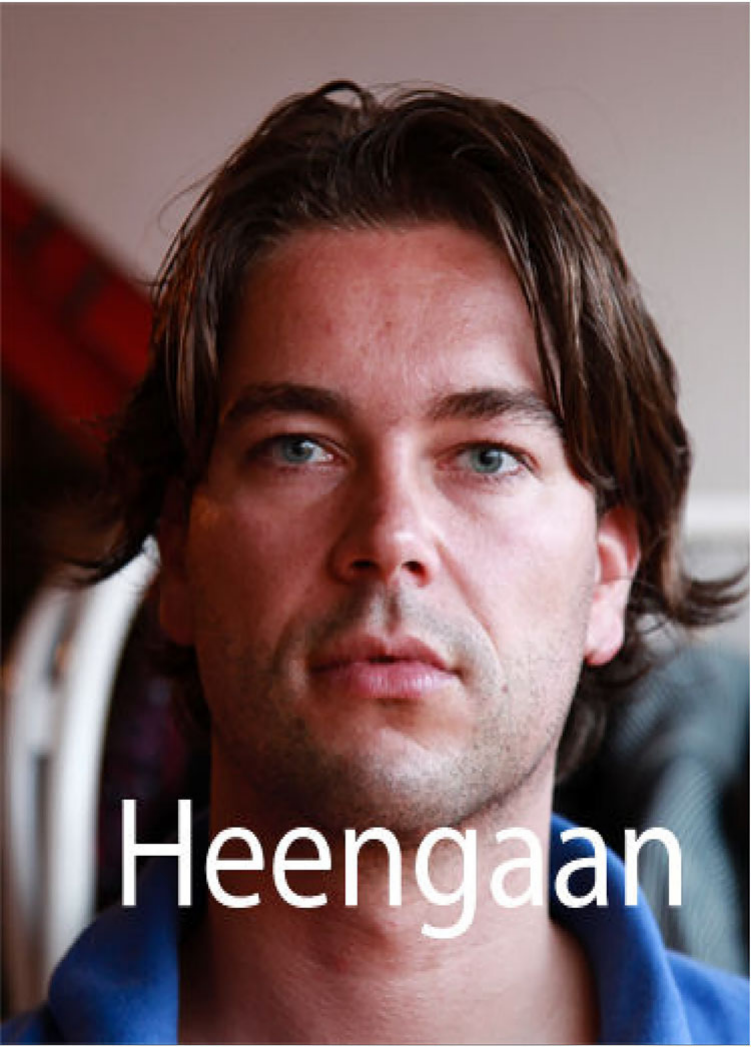
Example picture of a deceased person combined with a loss word *Note*: *Heengaan* is Dutch for “passing away.” The person in this picture is not deceased in real life and has provided written approval for the use of this picture for scientific publications.

For each participant, six pictures were used in this task: two portrait pictures of the deceased, two portrait pictures of a stranger matched to the deceased on age (age categories [in years]: 0–1, 1–5, 5–10, 10–15, 15–20, 20–25, 25–30, 30–40, 40–50, 50–60, 60–70, 70 or older) and gender identity (male, female; Eisma et al., 2014), and two stock landscape pictures. Pictures of the deceased were checked for quality and, if needed, adapted by removing parts of the picture so that the deceased would be in the center of the picture in a portrait format. All pictures were reformatted to 358 × 532 pixels at a 72‐dpi resolution. Additionally, we used six loss‐related words (i.e., *passing away*, *dying*, *loss*, *grave*, *death*, *parting*) and six neutral words (i.e., *line*, *terms*, *group*, *round*, *image*, *tendency*), matched in Dutch on word frequency and word length (Eisma et al., 2014), in the task. Five stimuli types were constructed by combining pictures and words, categorized as follows: picture deceased, picture stranger, picture deceased + loss word (i.e., loss reality reminder), picture stranger + neutral word, and landscape picture. Pictures of strangers were included to control for the presence of a person in the pictures. Picture stranger + neutral word stimuli were included to control for the addition of a word to a picture of a person, and landscape pictures were included to control for the mere presence of a color picture. Figure 1 shows a sample stimulus.

#### Computer task

To assess (a) approach toward the deceased and (b) the avoidance of loss reality reminders, we used a free‐viewing attention task. Participants were instructed that they should look at the pictures as if they were looking at a photo album. Once they had looked at any given picture as long as they preferred, they pressed the spacebar, after which the next picture would appear, until there were no more pictures to be shown. We used the viewing duration, in milliseconds, from the appearance of the picture until the pressing of the spacebar as a measure of attention for specific stimuli. All stimuli were presented on a 27‐in screen with a 1920 × 1080–pixel resolution. Stimuli were presented in a pseudorandom fixed order, as shown in Table 2.

**TABLE 2:** Viewing duration (ms) and reliabilities for 12 stimuli, in order of appearance

Picture type	*M*	*SD*	Reliability (ρ)^a^
Stranger 1	2,300	1,828	.41
Deceased 1	9,083	10,111	.88
Landscape 1	2,020	1,570	.69
Deceased 2	9,000	13,994	.88
Landscape 2	2,807	3,100	.69
Stranger 2	1,725	1,768	.41
Stranger 1 + neutral word	1,650	1,234	.80
Deceased 1 + loss word	7,568	16,089	.85
Landscape 1	1,845	1,498	.69
Deceased 2 + loss word	7,196	11,469	.85
Landscape 2	2,434	2,735	.69
Stranger 2 + neutral word	1,566	1,240	.80

^a^ Spearman's rho correlation between the first and second time a stimulus of a particular type (i.e., stranger, deceased, landscape, stranger + neutral word, or deceased + loss word) was shown (e.g., the correlation between the viewing time for Stranger 1 and Stranger 2 is the reliability for the stranger stimulus type).

### Measures

#### Sociodemographic and loss‐related characteristics

We used a self‐constructed questionnaire to assess sociodemographic characteristics (i.e., age, gender identity, and educational attainment) and loss‐related characteristics (i.e., time since loss, gender identity of the deceased, relationship to the deceased, cause of death, and expectedness of the death).

#### Prolonged grief symptoms

Prolonged grief symptoms were assessed using the 22‐item Traumatic Grief Inventory–Self Report Plus (TGI‐SR+: Lenferink et al., 2022). Participants rated the frequency of symptoms during the previous month on a 5‐point Likert scale ranging from 1 (*never*) to 5 (*always*). The sum score of all items was used as a general measure of prolonged grief symptom severity. Twelve items that correspond with the *ICD‐11* PGD criteria were summed to capture *ICD‐11* prolonged grief symptoms, and 10 items that correspond to the *DSM‐5‐TR* PGD criteria were summed to capture *DSM‐5‐TR* prolonged grief symptoms. A total score higher than 70 indicates clinically relevant levels of prolonged grief. Prior research has demonstrated support for the construct, convergent, and known‐groups validity of the TGI‐SR+ (Lenferink et al., 2022). In the present sample, reliability was excellent for the total scale, Cronbach's α = .95; *ICD‐11* items, Cronbach's α = .92; and *DSM‐5‐TR* items, Cronbach's α = .93.

#### Depressive symptoms

Depressive symptoms were assessed using the 16‐item Quick Inventory of Depressive Symptomology (QIDS; Rush et al., 2003; Dutch version: Lako et al., 2014). QIDS items are scored on a 4‐point scale ranging from 0 to 3, with varying anchors and higher scores indicating higher levels of depressive symptoms. Total scores are based on a scoring algorithm and range from 0–27, with scores of 6–10, 11–15, 16–20, and 21–27 indicating mild, moderate, severe, and very severe depressive symptoms, respectively. Past research supports the construct, convergent, and divergent validity of the QIDS (Lako et al., 2014). In the present sample, the QIDS demonstrated good reliability, Cronbach's α  = .82.

#### Lingering attachment

To assess lingering attachment (i.e., the extent to which participants still desired to use the deceased person as an attachment figure), we used the WHOTO (Hazan & Zeifman, 1994), which assesses three attachment‐related functions proposed by Bowlby (1982): proximity‐seeking (e.g., “Who is the person you most like to spend time with?”), safe haven (e.g., “Who is the person you want to be with when you are feeling upset or down?”), and secure base (e.g., “Who is the person you would want to tell first if you achieved something good?”). Participants were asked to list two to five people, in order of importance, in response to six questions, with two items per function. To assess lingering attachment, participants answered the following question for each item: “Even if you know you cannot or should not, if you could place your deceased loved one anywhere on the above list, where would you desire to put them?” Assigning the deceased to the first place resulted in a score of 5 for the item; assigning the deceased to the fifth place resulted in a score of 1 for the item. If the participant indicated that the deceased was not in the top five, 0 points were allocated for the item. We used scores from all six questions to calculate an average lingering attachment score (Fagundes, 2012). Several studies have provided evidence for the reliability and convergent, known‐groups, and test‐criterion validity of different versions of the WHOTO (e.g., Fagundes, 2012; Fraley & Davis, 1997). In the present sample, the WHOTO demonstrated excellent reliability, Cronbach's α  = .94.

### Data analysis

An a priori power analysis using G^*^Power3 (Faul et al., 2007) indicated that for a point‐biserial correlation using a one‐tailed test, with a medium effect size (*r*  = .30; Cohen, 1988) and an alpha of .05, a sample size of 64 participants was necessary to achieve a power of .80. We used TGI‐SR+ total score as a measure of prolonged grief symptoms in our main analysis. We calculated mean viewing durations for each stimulus type. We estimated test–retest reliability of the viewing duration per stimulus by calculating zero‐order correlations between viewing durations for the first and second stimuli of the same type.

Using correlation analyses, we tested our primary hypotheses that there would be (a) a positive association between prolonged grief symptom levels and viewing duration for pictures of the deceased and (b) a negative association between prolonged grief symptom levels and viewing duration for reminders of the loss reality. We also used correlation analyses to test our secondary hypotheses that there would be positive associations between lingering attachment and both prolonged grief symptom levels and viewing duration for pictures of the deceased. As the viewing duration variables had nonnormally distributed data, we employed nonparametric Spearman's correlations for all analyses that included these variables.

We conducted three sensitivity checks. First, we examined associations between viewing time for all control stimuli and both prolonged grief symptoms and lingering attachment. Next, we examined associations between depressive symptoms and viewing times for all stimuli types to assess if the effects were unique to prolonged grief symptoms. Lastly, we repeated the previously described main analyses using prolonged grief symptoms per *ICD‐11* and *DSM‐5‐TR* criteria instead of TGI‐SR+ total score to assess if the effects generalized across different criteria sets for prolonged grief symptoms. For exploratory purposes, we assessed the associations between sociodemographic and loss‐related variables and viewing times for all stimuli types (see Supplementary Materials).

We used two‐sided tests with a standard significance level (α = .05) for all analyses. Analyses were performed in IBM SPSS Statistics (Version 28). In line with guidelines by Cohen (1988), correlations between .10 and .29, .30 and .49, and higher than .50 were considered to indicate small, moderate, and large effects, respectively.

## RESULTS

### Preliminary analyses

#### Subsample comparisons

For descriptive purposes, we compared participants recruited through convenience sampling by students (*n* = 13) with those recruited through their participation in a previous survey study (*n* = 59) on all sociodemographic and loss‐related variables, lingering attachment, and symptom levels. The convenience sample was younger, *t*(70) = −.642, *p* < .001, *d* = −1.96; had experienced loss longer ago, *t*(70) = 3.62, *p* < .001, *d* = 1.10; and more frequently had lost a second‐degree (vs. first‐degree) relative (69% vs. 2%), *p* < .001 (Fisher's exact test). In addition, they reported lower lingering attachment, *t*(70) = −5.78, *p* < .001, *d* = −1.77; prolonged grief symptom levels, *t*(70) = −4.65, *p* < .001, *d* = −1.42; and depressive symptom levels *t*(70) = −2.82, *p* = .003, *d* = −1.11.

#### Symptom levels and correlations between predictors

The total sample showed nonclinical‐to‐clinical levels of prolonged grief symptoms (TGI‐SR+ total score: *M* = 44.53, *SD* = 16.91, range: 22–91) and depressive symptoms (QIDS: *M* = 5.89, *SD* = 4.40, range: 0–17). As shown in Table 3, prolonged grief symptoms were strongly and positively related to depressive symptoms and lingering attachment. *ICD‐11* and *DSM‐5‐TR* prolonged grief symptoms were also strongly and positively related to lingering attachment. *ICD‐11* and *DSM‐5‐TR* prolonged grief symptoms showed strong positive associations with depressive symptoms. Depressive symptoms and lingering attachment were moderately positively associated.

**TABLE 3:** Correlations between prolonged grief (PG) symptoms, lingering attachment, depressive symptoms, and average viewing duration (VD) for different stimuli types

Variable	PG symptoms (TGI‐SR+ total)	Lingering attachment	Depressive symptoms	*ICD‐11* PG symptoms	*DSM‐5‐TR* PG symptoms
VD deceased	.32^**^	.26^*^	.18	.34^**^	.31^**^
VD deceased + loss word	.14	.17	.09	.15	.12
VD Stranger	.09	−.06	.10	.04	.04
VD stranger + neutral word	.03	−.14	.08	−.04	−.04
VD landscape	.10	−.07	.15	.07	.07
PG symptoms (TGI‐SR+ Total)	–	.53^***^	.67^***^	.98^***^	.97^***^
Lingering attachment		–	.32^**^	.58^***^	.53^***^
Depressive symptoms			–	.63^***^	.63^***^
*ICD‐11* PG symptoms				–	.98^***^

*Note*: Spearman's correlations were used for analyses that included viewing times. Pearson correlations were used for analyses exclusively based on questionnaire scores. TGI‐SR+ = Traumatic Grief Inventory–Self Report Plus; *ICD‐11* = *International Classification of Diseases and Related Health Problems* (11th rev.); *DSM‐5‐TR* = *Diagnostical and Statistical Manual of Mental Disorders* (5th ed., text rev.). *p < .05 (two‐sided).

**p < .01 (two‐sided). ***p < .001 (two‐sided).

#### Means, standard deviations, and reliabilities of stimuli viewing duration

Table 2 shows the mean values, with standard deviations, of viewing duration for each stimulus, as well as the reliability estimates for each stimulus type. On average, participants looked for longer periods at pictures of the deceased, both alone and combined with a loss‐related word (*M* range: ∼ 7–9 s), compared to pictures of a stranger, pictures of a stranger combined with a neutral word, and landscape pictures (*M* range: ∼1.5–3 s). All viewing durations except those for pictures of a stranger, ρ(70) = .41, *p* < .001, showed acceptable‐to‐good test–retest reliability, ρ*s*(70) = .69–.88, *p*s < .001.

### Main analyses

Table 3 shows correlations between symptom measures and lingering attachment and average viewing durations for the five stimulus types. As predicted, higher prolonged grief symptom levels were related to a longer viewing duration for pictures of the deceased. However, prolonged grief symptom levels did not relate significantly to viewing duration for pictures of the deceased with a loss word (i.e., loss reality reminders). Higher levels of lingering attachment were also related to a longer viewing duration for pictures of the deceased but not viewing duration for loss reality reminders.

### Sensitivity checks

As a first sensitivity check, we examined whether prolonged grief symptom levels and lingering attachment were related to attention for control stimuli (i.e., picture stranger, picture stranger + neutral word, landscape picture), and no significant associations emerged, *p*s = .242–.833. As a second sensitivity check, we examined associations between depressive symptoms and the five stimuli types; again, no significant associations emerged, *p*s = .126–.521. As a third sensitivity check, we examined whether the effects of prolonged grief symptoms on attention held for prolonged grief symptoms as defined by the *ICD‐11* and *DSM‐5‐TR* criteria; all associations were equivalent in size, direction, and significance compared to analyses conducted with total TGI‐SR+ scores.

## DISCUSSION

The primary aim of this study was to test the approach avoidance processing hypothesis of PGD in an adult bereaved sample. Using a novel free‐viewing attention task, we aimed to establish whether higher levels of prolonged grief symptom severity were associated with (a) approach behavior toward the deceased and (b) avoidance of reminders of the loss reality. We found that prolonged grief symptom severity demonstrated a moderate positive association with voluntary attention toward the deceased. No significant associations emerged between prolonged grief symptom severity and voluntary attention toward loss reality reminders (i.e., picture of the deceased + loss word stimuli). The secondary aim of the study was to test whether lingering attachment to the deceased was related to prolonged grief symptom severity and voluntary attention toward the deceased: A stronger desire to use the deceased for attachment‐related needs was indeed related to higher prolonged grief symptom levels and a stronger attentional focus on the deceased.

The findings that severe prolonged grief symptom levels were related to stronger approach behaviors toward the deceased but not stronger avoidance of the reality of the loss align with some theories but not others. These findings support theories that propose a central role for approach tendencies toward the deceased in the development of severe grief reactions (e.g., Boddez, 2018; Kakarala et al., 2020; LeRoy et al., 2019). They do not support theories that propose that avoidance of the reality of the loss is a risk factor for prolonged grief (e.g., Boelen, van den Hout, et al., 2006; Shear et al., 2007). These results, therefore, only partly align with the approach avoidance processing hypothesis of PGD, which holds that behaviors characterized by approach behaviors toward the deceased and the avoidance of loss reality co‐occur among people with higher levels of prolonged grief symptom severity (Eisma & Lenferink, 2023).

When examining empirical research, the link between approach tendencies toward the deceased and prolonged grief severity observed in this study complements findings from surveys showing that behaviors focused on retaining a bond with the deceased predict increased prolonged grief symptom severity over time (e.g., Boelen, Stroebe, et al., 2006; Field et al., 2003). The lack of an association between prolonged grief symptoms and the avoidance of loss reality stimuli does not align with associations between avoidance strategies and prolonged grief symptom severity (for a review, see Eisma & Stroebe, 2021) nor the demonstrated effectiveness of exposure therapy, which counters such avoidance tendencies, in reducing prolonged grief symptoms (Boelen, van den Hout, et al., 2006; Bryant et al., 2014; Eisma, Boelen et al., 2015). It does, however, align with research showing mixed findings regarding the longitudinal predictive effects of loss‐related avoidance on prolonged grief symptoms (e.g., Boelen & Eisma, 2015; Eisma & Stroebe, 2021; Smith et al., 2024) and network analyses showing that loss‐related avoidance is not a central node in prolonged grief symptom networks (e.g., Robinaugh et al., 2016).

A consideration of the results of the only previous study investigating the approach avoidance processing hypothesis may help to clarify the present results. Eisma and Lenferink (2023) used latent class analysis to demonstrate that bereaved adults with the highest odds of engaging in behaviors that appear to maintain a focus on the deceased and behaviors indicative of loss‐reality avoidance (vs. those with lower odds of these behaviors) showed the highest levels of prolonged grief symptom severity and highest odds of probable PGD. However, the findings also showed the existence of a group of participants showing high odds of approach behaviors and low odds of avoidance behaviors, who, on average, demonstrated subclinical levels of prolonged grief symptoms and lower odds of probable PGD. In the present study, the latter subpopulation of bereaved adults may have been overrepresented in the predominantly subclinical sample, explaining the pattern of findings.

Compared to previous laboratory studies, the present study is one of the first to demonstrate convincingly that people with higher levels of prolonged grief symptom severity show stronger approach behaviors toward the deceased. Somewhat similar results were reported in a previous AAT study showing that bereaved adults with high levels of prolonged grief symptoms were faster in pulling the name of a deceased loved one toward themselves than in pushing the name away (Maccallum & Bryant, 2019). However, in that study, similar patterns were observed for names of other attachment figures and neutral names. Notably, another study in which an AAT was performed by people who had experienced the dissolution of a romantic relationship similarly demonstrated positive associations between approach bias toward one's ex‐partner and both breakup grief severity and lingering attachment (Eisma et al., 2022). However, the AAT assesses automatic approach and avoidance tendencies, making these findings difficult to compare with the present study, which focused on voluntary attention. The present study is also one of few studies on attention in bereaved adults to use stimuli that represent the loss reality (i.e., a picture of the deceased plus a loss‐related word). Most previous studies have used generic loss‐related stimuli, thereby precluding conclusions regarding the association between prolonged grief symptoms and avoidance of the loss reality. Notable exceptions to this are two studies—one that used AAT and one that used an eye‐tracking task—that demonstrated associations between grief‐related rumination and both automatic and voluntary avoidance of the loss reality (Eisma et al., 2014; Eisma, Rinck, et al., 2015). Complementing the null findings on prolonged grief symptom severity and avoidance of the loss reality in the present study, prolonged grief symptoms did not predict avoidance of the loss reality over and above grief‐related rumination in either of these studies.

This study also provides information about the psychometric properties of our free‐viewing attention task. Viewing durations for all stimuli types except pictures of strangers showed acceptable test–retest reliability. It is possible that a subset of participants responded more slowly to the first presentation of a picture of a stranger because it was the first stimulus shown to all participants, thereby reducing test–retest reliability. We advise incorporating practice trials with neutral pictures, including pictures of strangers, in the next version of this task. The comparability of our main effects across different laboratory tasks, discussed in the previous paragraph, provides some evidence for the construct validity of the task. Nevertheless, further research on the validity of this new attention task is warranted.

Clinically, the findings suggest the usefulness of addressing the potential role of approach behaviors toward the deceased in reducing prolonged grief symptoms. Existing therapeutic approaches for prolonged grief, such as CBT, already include techniques focused on reducing persistent and severe continuing bond behavior (Boelen et al., 2007; Boelen, van den Hout, et al., 2006). A complementary therapeutic approach could be behavioral activation (Eisma, Boelen, et al., 2015; Papa et al., 2013), which fosters adjustment to life without a loved one, generates positive emotions, and distracts from negative thoughts and feelings.

We also explored the associations between lingering attachment and both approach and avoidance tendencies and prolonged grief symptoms. A stronger desire to use the deceased as an attachment figure was positively related to prolonged grief symptoms and attention toward the deceased. Lingering attachment represents an inability to rearrange one's attachment hierarchy so that other people in their social environment meet the attachment‐related needs previously fulfilled by the deceased. The continued desire to be close to the deceased and use them as a source of safety and security is theorized to interfere with psychological and physiological adaptation to loss (LeRoy et al., 2019; Mikulincer & Shaver, 2022); that is, people with difficulties adapting their attachment hierarchies may continue to experience a desire to reconnect with the deceased, grief‐related rumination, and recurring distress due to their inability to reunite (Eisma et al., 2022; LeRoy et al., 2019). Clinically, these findings suggest that a reduction in the desire to use the deceased for attachment‐related needs while simultaneously making meaningful connections with other people or deepening existing social relationships with close others may help reduce prolonged grief severity.

Some limitations warrant mention. First, the sample predominantly consisted of middle‐aged women from Western countries who reported higher levels of educational attainment. It remains to be established whether the findings are generalizable to samples that include more men, individuals with lower levels of educational attainment, and people with different cultural backgrounds. The latter is particularly pertinent because loss‐related avoidance tendencies may differ between cultures (Bonanno et al., 2005). Second, prolonged grief symptom levels ranged from nonclinical to clinical. Though a broad range of scores is important for detecting correlations, as it prevents a restriction of range, people with more severe prolonged grief symptoms or a PGD diagnosis may show stronger approach and avoidance tendencies. Therefore, associations reported in this study could be more pronounced in a sample of individuals with higher symptom levels. It is important to compare people with and without PGD on this task in future research. Third, we examined attention toward easy‐to‐standardize static visual facial stimuli; future research should explore the application of other stimuli types to study attention in this area, such as videos or voice recordings of the deceased. Fourth, our analyses were limited to examining bivariate concurrent associations between the constructs of interest; the study design and modest sample size precluded the exploration of more complex associations, such as mediation or moderation effects. Unraveling the temporal associations between loss‐related characteristics (e.g., kinship), lingering attachment, approach and avoidance tendencies, and prolonged grief symptoms remains an important goal for future research.

Despite these limitations, the present study is one of the first to test the approach avoidance processing hypothesis using a behavioral measure. Using a novel free‐viewing attention task, we demonstrated that more severe prolonged grief symptoms were associated with stronger approach tendencies toward the deceased. Moreover, lingering attachment to the deceased was related to higher levels of prolonged grief symptom severity and stronger approach tendencies toward the deceased. The findings suggest that approach behavior toward a deceased loved one are characteristic of people experiencing more severe grief reactions. Clinically, the findings suggest the usefulness of reducing severe and persistent continuing bonds behaviors, while encouraging engaging in new social relationships or deepening existing social connections, in the treatment of prolonged grief.

## OPEN PRACTICES STATEMENT

The study reported in this article was not preregistered. Raw data, processed data, syntax, and output is available via https://doi.org/10.34894/JAZGWX

## AUTHOR NOTE

Maarten C. Eisma was supported by a Veni grant of the Dutch Research Council (NWO; Grant ID: 016.veni195.113).

The funder did not play a role in the study design; collection, analysis, or interpretation of the data; writing of the report; or decision to submit the article for publication.

## Supporting information



Supporting Information
