# Supplementary material for: Prolonged grief symptoms and lingering attachment predict approach behavior toward the deceased
Source: J Trauma Stress. 2025 Jan 6;38(2):284–95. doi: 10.1002/jts.23124 (PMC11967323; doi:10.1002/jts.23124)
Supplement: Supplementary file 1 — Supporting Information [file JTS-38-284-s001.docx]

**Supplementary File A**

We explored the associations between all sociodemographic and loss-related variables and average viewing times for all stimuli types. Most of these variables (education level, time since loss, cause of death, expectedness death, gender deceased) were not associated with average viewing times for any stimulus type (all *p's* > .05).

However, males (vs. females and other gender) showed shorter viewing times for pictures of strangers, *z* = -2.04, *p* = .041, pictures of strangers with neutral words, *z* = -2.40, *p* = .016, and pictures of the deceased + loss words, *z* = -2.87, *p* = .004. Similarly, people who lost other relationships (vs. first-degree relationships) showed shorter viewing times for pictures of landscapes, *z* = -2.00, *p* = .045, pictures of the deceased, *z* = -.2.57, *p* = .010, and pictures of de deceased + loss words, *z* = -3.159, *p* = .002. Those who were younger showed shorter viewing times towards pictures of the deceased, ρ(70) = .37, *p* < .001, and pictures of the deceased + loss words, ρ(70) = .39, *p* < .001.

These findings should be interpreted with caution. Only the correlations of age with viewing times for the deceased and deceased + loss words remained significant, after applying a Bonferroni correction for the 40 comparisons conducted as part of these exploratory analyses (α = .05/40 = .00125). Some compared subgroups were small (e.g., *n* = 10 had experienced bereavement of second-degree family member, *n* = 12 were male). Those who lost a second-degree family member (vs. first-degree family member) were much younger, *z* = -3.86, *p* < .001, suggesting that effects of age were partially driven by kinship with the deceased.
